# Progress and Prospective of the Industrial Development and Applications of Eco-Friendly Colorants: An Insight into Environmental Impact and Sustainability Issues

**DOI:** 10.3390/foods12071521

**Published:** 2023-04-03

**Authors:** A. Annam Renita, Tejal K. Gajaria, S. Sathish, J. Aravind Kumar, D. Shanthana Lakshmi, Joanna Kujawa, Wojciech Kujawski

**Affiliations:** 1 Department of Chemical Engineering, Sathyabama Institute of Science and Technology, Chennai 600119, India; 2 Division of Biomedical and Life Sciences, School of Science, Navrachana University, Vadodara 391410, India; 3 Department of Energy and Environmental Engineering, Saveetha School of Engineering, SIMATS, Chennai 600119, India; 4 RSK Environment Ltd., 18, Frogmore Road, Hemel Hempstead HP3 9RT, UK; 5 Faculty of Chemistry, Nicolaus Copernicus University in Toruń, 7 Gagarina Street, 87-100 Toruń, Poland

**Keywords:** natural colorants, extraction techniques, technological applications, environmental sustainability, colorant stability, nutraceuticals

## Abstract

Color is the prime feature directly associated with the consumer’s attraction and choice of their food. The flavor, safety, and nutritional value of any food product are directly associated with the food color. Natural and synthetic colorants (dyes and pigments) have diversified applications in various sectors such as food, feed, pharmaceutical, textiles, cosmetics, and others. Concerning the food industry, different types of natural and synthetic colorants are available in the market. Synthetic food colorants have gained popularity as they are highly stable and cheaply available. Consumers worldwide prefer delightful foodstuffs but are more concerned about the safety of the food. After its disposal, the colloidal particles present in the synthetic colorants do not allow sunlight to penetrate aquatic bodies. This causes a foul smell and turbidity formation and gives a bad appearance. Furthermore, different studies carried out previously have presented the toxicological, carcinogenic effects, hypersensitivity reactions, and behavioral changes linked to the usage of synthetic colorants. Natural food colorings, however, have nutraceutical qualities that are valuable to human health such as curcumin extracted from turmeric and beta-carotene extracted from carrots. In addition, natural colorants have beneficial properties such as excellent antioxidant properties, antimutagenic, anti-inflammatory, antineoplastic, and antiarthritic effects. This review summarizes the sources of natural and synthetic colorants, their production rate, demand, extraction, and characterization of food colorants, their industrial applications, environmental impact, challenges in the sustainable utilization of natural colorants, and their prospects.

## 1. Introduction

Colorants are key to the acceptance of various commodity materials as they provide wider perspectives from sensory perceptions. They have diverse roles during the lifespan of mankind, ranging from textiles to the colors of contact lenses. The significance of colorants has an immense impact on commodity markets. Hence, understanding the sources of colorants is highly significant in a few areas, such as the textile and food industries. It brings paramount importance to the study of biochemistry, processing, and characterization of colorants, irrespective of their sources; natural or synthetic. Based on their solubility, they can be classified as soluble colorants, i.e., dyes, and insoluble colorants, i.e., pigments. Dyes are often believed to have a synthetic origin. Despite their natural origin, pigments have limited utilization as a colorant in many industries; the details are summarized in the respective upcoming sections, making synthetic or semi-synthetic dyes a potential source in our routine life. However, many dyes, pigments, or synthetic colorants show potential toxicity to humans, which warrants a regulatory mechanism to ensure their controlled and safe usage. The initial attempt to safeguard the utility of food colorants was brought about by the Food and Drug Act of 1906 in the United States of America (USA). The first international food standards and safety guidelines are called Codex Alimentarius, established in 1962. 

**Figure 1:**
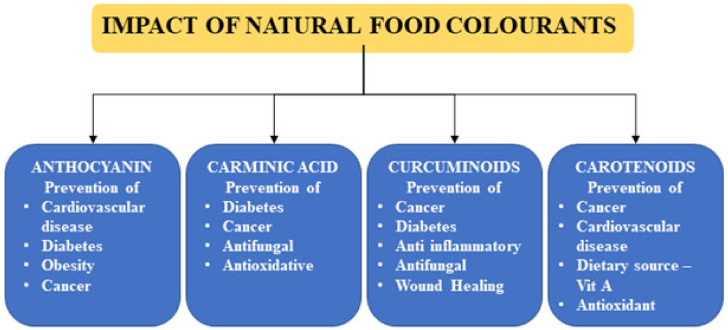
Beneficial effects of natural food colorants on human health.

The Codex Alimentarius functions as the Codex Committee on Food Additives and Contaminants (CCFAC). This ensures that it complies with the established food standards and safety guidelines. CCFAC regulates additives and colorants in food and allied products. A database regarding commercially utilized and other food additives’ biological activities has been maintained online as a joint effort of Codex Alimentarius and WHO/FAO; this is called the general standards for food additives. Such inventories/databases are critical during international trade among different nations that might have differences of opinion regarding the usage and permissible levels of food additives. Many countries have adopted their permissible levels as recommended by Codex Alimentarius. Besides, a few countries, such as the European Union (EU), Korea, India, Japan, and USA, have legislation to ensure permissible levels of food additives. Despite being natural in origin, natural colorants have significant concerns as food additives due to their sensitivity to heat, light, and oxygen, which results in alteration in their color or flavor. If utilized under such circumstances, it deteriorates their properties of being beneficial to human health [1]. Beneficial effects of natural colorants are illustrated in Figure 1. A brief discussion of natural coloring agents, their origin, and classification along with their chemical and biological features was exclusively performed. A few modern extraction techniques for natural coloring agents and their associated dyeing mechanisms are also discussed herein. A selective coloring agent must possess better capacity on the substrate components, which must be economically viable as well as biodegradable. Despite posing several beneficial characteristics of non-toxicity, biodegradability, and extraction easiness, few coloring components comprise medicinal values, which make them suitable candidates for providing colors. Though they possess such benefits, they are economically imbalanced owing to reduced yield and lower performance. Owing to its environmentally benevolent nature, and lower drudging production cum extraction technique, the naturally made coloring agents have emerged as effective replacements for toxic synthetic colorants.

The present review is intended to reveal the latest developments in food colorants. It provides specific details regarding natural and synthetic colorants routinely employed in food industries, their biochemistry, sources, and applicability on various legislations, as mentioned earlier. A brief note is provided about the advancements in extracting and purifying natural colorants. Furthermore, this review presents the emerging challenges in using natural colorants in the industrial sector. The article concludes with a prompt discussion about the future prospects for the possible utilization of colorants in their diversified applications.

## 2. Natural Colorants and Their Classification by Source 

### 2.1. Origin-History

The usage of colorants came into being from the ancient stone ages and even after the growth of the weaving practice, the involvement of dyes was expanded to textile units in various civilizations. Ancient common dyes include blue indigo, madder, and yellow dye availed from turmeric or saffron, whereas limestone, ochre, and charcoal were treated as important coloring agents [2]. The major origin of naturally made colorants comprises minerals, plants/animals, and almost all parts of plant varieties such as roots, leaves, flowers, fruit, wood, seed, and bark were utilized to obtain several colors along with their pertinent combinations. In the olden stone ages, minerals of several colors were predominantly utilized as pigmenting agents whereas in Phoenicians, ancient Roman/Indian, Egyptian, and African regions mineral components were utilized as bio-coloring agents [3].

**Figure 2:**
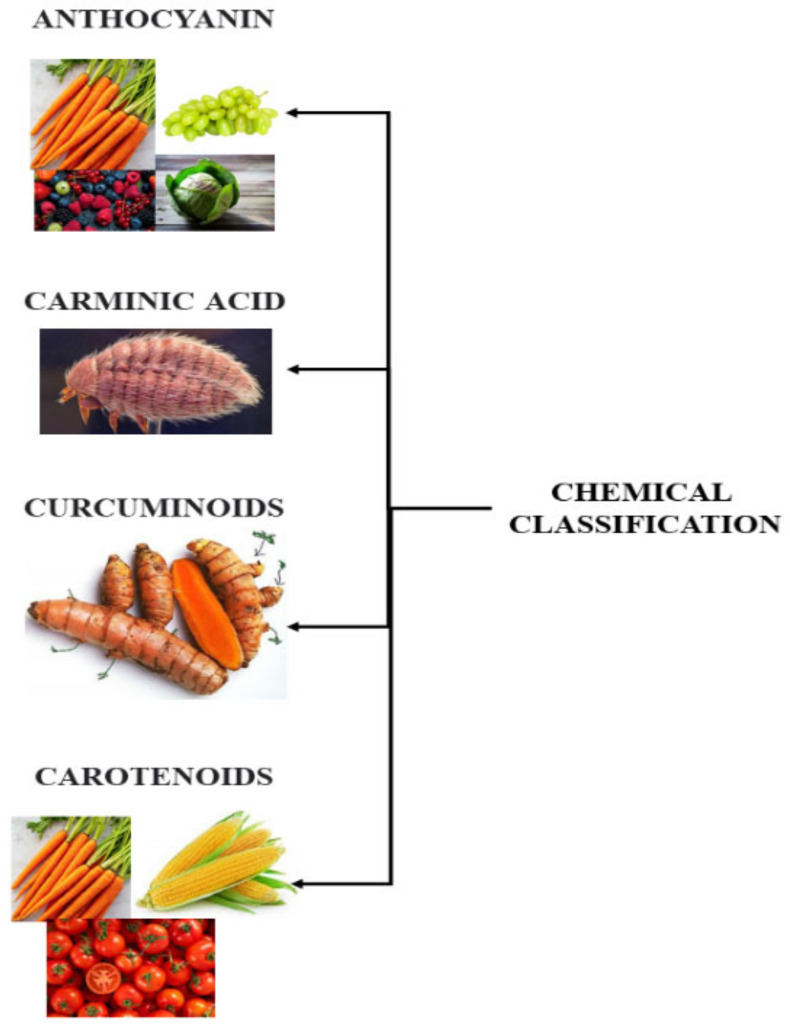
Types of food colorants obtained from natural sources.

Natural colorants possess various beneficial features that make their classification complex. There are wide varieties of natural sources from which natural colorants are derived. Hence, each colorant has a unique chemical composition that influences its properties of solubility, stability, heat sensitivity, and pH [4]. Some of the natural sources of food colorants are given below. Furthermore, colorants obtained from natural sources are illustrated in Figure 2.

### 2.2. Classification by Source

#### 2.2.1. Plants

Plant-based colors are produced in plants due to biochemical pathways that take place within them. Hence, the colored compounds have unique physicochemical properties. Since different natural organic-colored compounds are available from plants, they play a significant role in photosynthesis and pollination [5]. There are many colorants obtained from plants, of which curcumin is a natural yellow-colored powder which is described in detail below. Curcumin (1, 7-bis-(4-hydroxy-3-methoxyphenyl)-1,6-heptadiene-3,5-dione or diferuloyl-methane) is acquired from the plant *Curcuma longa l*—the rhizome of the plant, abundantly grown in India. It is also known as diferuloylmethane, a low-molecular-weight, natural, and polyphenolic compound. Curcumin belongs to the ginger family and is primarily grown in the tropical and sub-tropical parts of the world. It is also called yellow-saffron or turmeric yellow and has wide applications as a food colorant. Besides being a food colorant, it has great flavoring properties. There is very little toxicity and no genotoxicity associated with curcumin. The acceptable daily intake of curcumin is 15.75 mg/kg body weight. Curcumin is economical with excellent antibacterial, antiviral, antifungal, antitumor, antioxidant, and anti-inflammatory properties. Hence, it is used in a broad range of food products, such as curry, milk and milk products, jams, jellies, juices, chewing gums, seafood, and other snacks [6,7].

As mentioned earlier, curcumin is effective against a vast number of human diseases. In order to evaluate the efficacy of curcumin against human ailments, more than 65 trials in humans have been performed. Besides, 35 clinical trials in humans are in progress. Curcumin’s biological activities are majorly attributed to bis-alpha, beta-unsaturated beta-diketone, two methoxy groups, two phenolic hydroxy groups, and two double-conjugated bonds present in the curcumin. In spite of the advantages of curcumin, some of the factors, such as low solubility in aqueous solution and poor bioavailability, limit the nutraceutical usage of curcumin [8]. 

Furthermore, the molecular structure of curcumin changes under different pH conditions. These effects were attributed to changes in the molecular structure of curcumin under different pH conditions. There was little modification in the color of curcumin-loaded emulsions when stored under acidic conditions, but their yellow color diminished when stored under alkaline conditions. Furthermore, as discussed above, curcumin has excellent antimicrobial properties against pathogens such as *Staphylococcus aureus, Listeria monocytogenes*, *Escherichia coli*, and other cultures of fungi, *Penicillium* spp. and *Candida albicans*. This makes it a potential agent in intelligent or active packaging besides being a food colorant and flavoring agent [9,10]. This capacity can have a weakening impact on curcumin-containing items. Then again, light-initiated oxidation can be applied in frameworks with horrendous organic behavior, for example, in the killing of microscopic organisms.

#### 2.2.2. Animal

Animals and plants contribute to producing different organic-colored compounds with diverse chemical compositions. Colorants obtained from animals have numerous roles, such as the transportation of oxygen in the blood, protection from UV rays, and much more. Many colorants are derived from animals, such as purines, melanin, and carminic acid [11]. Among the above colorants, carminic acid, a widely used red food colorant, is briefed in the section below. Certain insects, such as cochineal *Coccus cacti* L., possess carminic acid, a red-crimson anthraquinone coloring matter. Hydroalcoholic extraction of insect bodies yields cochineal or cochineal extract. Carmine contains about 50% carminic acid, and its derived pigments are utilized to provide red shades in alcoholic beverages, ice creams, and confectionaries [12]. Though the colorant does not cause adverse effects, it rarely produces allergic reactions or anaphylactic shock. Carmine has excellent thermal and light stability features. Though carmine was initially used only in the cosmetics and textile industries, later, it found application in the food industry as a natural colorant. Carmine can be classified as carminic acid and carminic aluminum lake. Carminic acid’s photo activity property helps it exhibit different colors in different environments. This enables carminic acid to be used in diversified food types. For instance, the color of the acid changes from orange to purple with an increase in pH from 2 to 12. Besides, the color changes to taupe on its exposure to Fe^3+^ or Ca^2+^. Further, the color changes to purple after being exposed to protein. The commonly employed food colorant is a carminic aluminum lake, formed by the aluminum atom being chelated with the help of two molecules of carminic acid through the 5-hydroxyl group and ortho-carbonyl oxygen. Although the color of the carminic aluminum lake is found to be more stable when compared with that of carminic acid, the safety features of aluminum are a concern regarding its use in food products [13]. 

#### 2.2.3. Microbes and Minerals

Colorants are also produced from microbes; carotenoids and *Monascus* pigments are commercially and economically beneficial as we can control the growth conditions of the microbes. This has a greater future scope and will be elaborated on in the upcoming section [14]. Minerals are often formed as a result of a geological process, and they are either chemical compounds or elements. Depending on the nature of the mineral source, colorants obtained from them vary in their chemical composition as well as they possess unique physical structures

## 3. Classification Based on Their Chemical Structure

### 3.1. Anthocyanins: Flavonoid Derivatives

Flavonoids are a gathering of optional plant metabolites portrayed by a C_6_C_3_C_6_ carbon skeletal spine of these polyphenolic compounds. Anthocyanins are a significant subclass of water-dissolvable shades that bestow distinctive red-to-blue tones on plants [15]. Six significant aglycones (anthocyanidins) are found in regularly burned-through soil products, varying in level of hydroxylation and methoxylation. These aglycones are undoubtedly linked to sugars and may be further acylated with sweet-smelling or aliphatic acids. Both glycosylation and acylation improve the anthocyanin soundness, representing that basic anthocyanidins are seldom found in nature [16]. Anthocyanins account for the largest proportion of water-dissolvable naturally occurring colors; more than 700 remarkable constructions have been recognized [17]. The utilization of anthocyanins as regular food shades has experienced hardships because of poor stability; the shade of anthocyanins is sensitive to light, heat, oxygen, and pH conditions, which restricts their utilization in various food items [18]. Along these lines, the food industry searches for techniques to improve anthocyanin stability.

Studies have shown that the shade of anthocyanins can be settled and fortified by co-pigmentation communications with dry particles in arrangement with the color. Co-pigmentation is evidenced by shifts in the frequency of greatest assimilation or expansions in the force of absorbance [19,20,21]. The connection system is believed to be the consequence of hydrophobic associations or π-π stacking [22]. Acylation of the anthocyanin is additionally thought to influence the shading articulation through intramolecular co-pigmentation [20]. Acylated anthocyanins are more common to vegetable and botanical frameworks, though non-acylated anthocyanins are transcendent in natural products. The unrivaled soundness of acylated anthocyanins under many conditions common in food applications clarifies vegetable-based anthocyanin colorants’ prevalence in the food industry [23,24].

The sensitivity of anthocyanins to pH considers interconversion between various red and blue primary structures and improving purple tones. As pH is expanded from acidic conditions, the flavylium cations (which, as a rule, seem red, pH ≤ 3) become deprotonated, losing shading (pH 3–6), and afterward, at last, shaping quinoidal bases (which seem purple-blue; pH ≥ 6). As a result of these immediate changes as a reaction to pH, anthocyanins regularly seem red or, to a great extent, vapid in the somewhat acidic pH conditions of numerous food items. An extra extension of the shading articulation of anthocyanins can likewise be an aftereffect of metal particle chelation by color, a component frequently seen in flower frameworks. Blue movements can be prompted uniquely in anthocyanins with catechol or pyrogallol moieties on the B-ring. Metal particles uproot hydrogen particles, initiating a change of flavylium cations (red) to quinonoid bases (blue) and afterward organizing stacking of the shade with another anthocyanin atom.

### 3.2. Isoprenoid Derivatives: Carotenoids

Carotenoids, one of the groups of natural pigments [25], are lipid-soluble, yellow–orange–red pigments found in all higher plants and some animals. Animals cannot synthesize carotenoids, so their presence is due to dietary intake. The most important carotenoids are carotenes (alpha-carotene, beta-carotene, beta-cryptoxanthin, lutein, and lycopene) and xanthophyll, including violaxanthin, neoxanthin, zeaxanthin, and canthaxanthin [25,26]. ß-Carotene is orange-yellow in color and oil soluble but can be made into a water-dispersible emulsion. Carrot (*Daucus carota*) is a good source of ß-carotene [27]. However, most ß-carotene for commercial use is now derived from algae. Oil palm [25,28], orange, apricot, mango, peach, and pepper contributed significantly to increasing ß-cryptoxanthin and ß-carotene concentrations of foods. Besides being used as colorants, carotenes are also used for nutritional purposes as provitamin A agents, as in margarine, where they also provide color, or as dietary supplements [28]. Saffron is water soluble and considered the most expensive colorant and spice [29,30]. The flower is light purple with a thread-like red stigma, which is a pigment. The odor of saffron is sometimes described as sea air to express its color shade and fragrance. The color appears as a powerful yellow one that finds its applications in saffron rice [31]. The phytopigment crocin is also found in Cape jasmine or gardenia, *Gardenia angusta*, fruits [28].

### 3.3. Pyrrole Derivatives: Chlorophyll

Chlorophyll pyrrole is a five-membered ring with four carbon atoms and one nitrogen atom. They are highly employed in forming hydrogen bonds and coordinating metals. Widely used pyrrole derivatives are heme and chlorophyll pigments. The structure of chlorophyll includes a porphin ring with a phytol attachment and a central magnesium ion. The phytol attachment and the magnesium ion make the chlorophyll pigment applicable to the food industry as a colorant. The phytol attachment is separated upon alkaline saponification, which alters the chlorophyll from lipophilic to hydrophilic. Hydrogen ions displace the magnesium ion when the chlorophyll pigment is treated using a weak acid. This brings about the formation of pheophytin, which is indicated by employing drab olive-green colors. When Cu^2+^ or Zn^2+^ ions are added to the center of the structure, it adds stability to the pigment and causes the expression of other useful green colors. The above complex formation can occur naturally or be induced after chlorophyll pigment is extracted [32].

### 3.4. Nitrogen-Heterocyclic Derivatives: Betalains

They are yellow and red colored pigments that are derivatives of betalamic acid. They consist of red-violet betacyanins and yellow-orange betaxanthins. They are water-soluble and found in plants from the order Caryophyllales, red beet, and cactus pear. They have good stability within a pH ranging from 3 to 7, and their color is not dependent on the food product’s pH value when compared with the rest of the natural food colorants. Hence, in addition to anthocyanins, betalains are added to food products with low acidity and neutral foods. Though betalains are independent of the pH of food products, other factors, such as temperature, light, and oxygen water activity, affect their stability. A yellow-colored betalamic acid is formed when hydrolytic cleavage occurs at the aldimine bonds under higher values of pH as well as during external heat application. This feature decreases the strength of the colorant resulting in limited shelf-life. External agents are added to increase the stability of betalains. For instance, pectin, locust bean gum, citric acid, and ascorbic acid are found to increase the stability of betalains [33,34].

### 3.5. Phycobiliproteins

**Figure 3:**
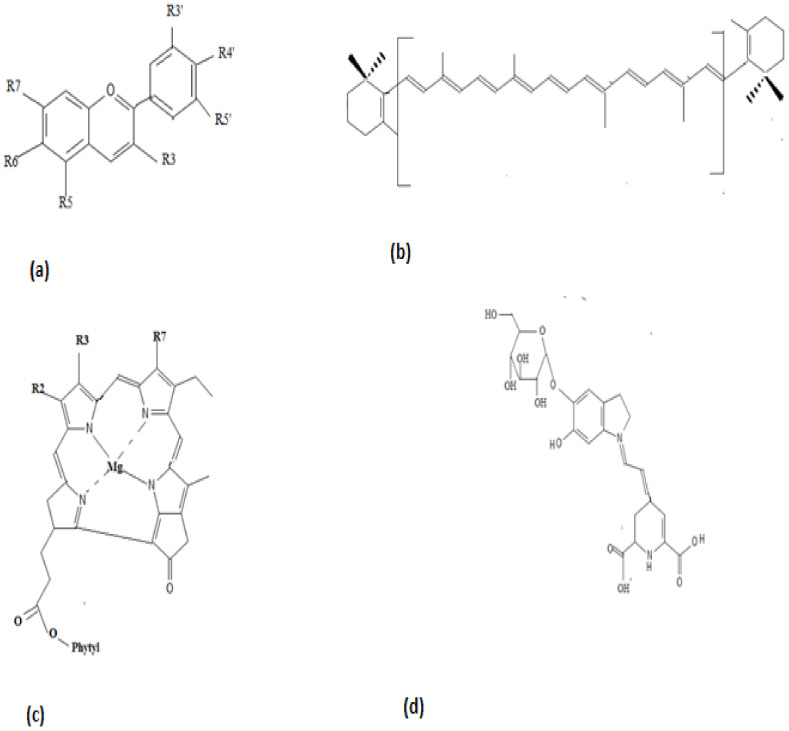
Chemical Structure of (**a**) anthocyanin, (**b**) β-carotene, (**c**) chlorophyll, and (**d**) betalain.

Phycobiliproteins serve as natural protein colorants in the food and cosmetic industry. Phycocyanin, extracted from *Spirulina platensis,* is a natural colorant in chewing gums, dairy products, and jellies. It exhibits a bright blue color and finds its application as a colorant in fermented milk products, ice creams, soft drinks, desserts, sweet cake decorations, milkshakes, and cosmetics. While the color of the pigment does not alter with pH or light, it is found to be sensitive to heat. For example, the pigment is stable for 40 min at 60 °C within a pH ranging between 4 and 5. As it is stable in acidic conditions, the pigment can be applied in confections and beverages such as Pepsi. In the case of Pepsi, the color lasts for one month while kept at room temperature. Furthermore, the color of the pigment has good stability in dry preparations; hence, sugar flowers used in the cake can maintain their colors for years [34]. The chemical structures of anthocyanin, β-carotene, chlorophyll, and betalain are illustrated in Figure 3.

## 4. Artificial Colorants

They are compounds that do not exist in nature and are produced by chemical synthesis. Coal tar is the source of the first artificial color, purplish lilac, discovered by William Henry Perkin in 1856. Artificial colorants possess numerous advantages, such as high coloring ability, stability, homogenous color tone, and the availability of various colors. Furthermore, they are easy to apply compared to natural colorants [35]. Depending on their water solubility properties, they are classified into three categories in the sections below. 

### 4.1. Water-Soluble Colorants

They are generally soluble in water, and some of the colorants include amaranth, Allura Red, tartrazine, and erythrosine.

Ponceau 4R, an azo dye, is used as a food colorant that imparts red when added to soft drinks, sweets, beverages, and other bakery products. The proposed ADI for Ponceau 4R is 0.7 mg/kg/bw/day. Ponceau 4R, sunset yellow, and tartrazine contribute around 65% of the commercial colorants market. Though it has been widely used in food products, there are certain side effects such as mutagenesis, reproductive toxicity, neurotoxicity, and headache when consumed more than the ADI. Further, it is believed to contain a potential carcinogen. Hence, strict regulations are proposed globally on the usage of the colorant. Moreover, it is banned in countries such USA, Canada, Norway, and Finland. Though techniques such as high-performance liquid chromatography, capillary electrophoresis, and spectrometry are available for the detection of Ponceau 4R, the development of rapid as well as sensitive methods is required to enhance the food quality as well as food safety [36,37].

Azorubine, also known as carmoisine, is a red colorant that is used in food products and drugs [36]. The ADI mentioned for carmoisine is 0–4 mg/kg body weight/day. It is used as a complement to materials that are being used for life science purposes. The colorant may be converted to aluminum lakes, which in turn causes exposure to aluminum. The maximum permitted level of the colorant is 500 mg/kg of food for decorations, coatings used in food products, sauces, and other related products. Further, it can be used in non-alcoholic drinks (50 mg/L) and spirituous beverages (200 mg/L). This colorant does not cause any side effects relating to genotoxicity, reproductivity, and carcinogenicity. Furthermore, behavioral changes in children have not been noticed when compared with the other colorants. Hence, the EFSA revised the ADI to 4 mg/kg/bw. However, hypersensitivity reactions are observed in some studies even when the colorant is used at a level lower than the ADI [38].

Allura Red is one of the engineered food colors used in sodas, yogurts, meat, and other bakery items. The permissible ADI is 7 mg/kg bw. Similar to other azo dyes, Allura Red is converted to aluminum lake due to the reaction of aluminum oxide with the coloring ingredient. Allura Red and the food products reach the gastrointestinal tract and the circulatory system. This, in turn, combines with the proteins during its vehicle and digestion process. Overconsumption of Allura Red causes adverse effects such as hypersensitivity, allergies, cancer, multiple sclerosis, food intolerance, lack of attention, cardiac disease, asthma, nausea, and brain damage. This is because of the reaction of the aromatic azo compounds. Hence, most countries have imposed strict regulations and banned the usage of Allura Red [39].

### 4.2. Oil Soluble Colorants

They are not soluble in water as they do not have any groups that can form salts, as in the case of water-soluble colorants. Oil-soluble colorants are highly toxic and are not used in the food industry. A previous study mentioned that using an oil-soluble colorant, Penso SX, for coloring butter and margarine was banned in 1976 [40,41].

### 4.3. Lake Colorants

They are neither soluble in water nor oils. They are formed from fine powders and are water-insoluble precipitation of aluminum hydrate. The dye content and particle size determine the product’s color. They are usually dispersed in food products and find their application in cakes, biscuits, confectionary, powdered drinks, and soups [42].

## 5. Extraction and Characterization of Natural Colorants

### 5.1. Anthocyanins

Anthocyanins are one of the principal natural pigments in the plant realm. They belongs to the flavonoid class of polyphenols. Anthocyanins are polyhydroxy derivatives of 2-phenyl benzo pyrylium. Literature shows that more anthocyanins have been distinguished and enlisted. Classification of anthocyanins is based on the amount and position of the hydroxyl groups, sugar groups, degree of methylation, and the presence of functional groups linked to sugar entities. The most common anthocyanins are cyanidin, peonidin, pelargonidin, delphinidin, petunidin, and malvidin. Among these, cyanidin, delphinidin, and pelargonidin are non-methylated anthocyanins. Many researchers for the recovery of anthocyanins discussed conventional techniques such as solvent extraction. Anthocyanins are extracted from *Hibiscus sabdariffa* flowers [43] by ultrasound-assisted extraction, and butterfly pea (*Clitoria ternatea* L. Flowers) by solvent extraction using ethanol as a solvent. Furthermore, anthocyanins are extracted from red cabbage [44], black rice (*Oryza sativa* L.) [45], black currants [46], purple sweet potato [47], cabernet sauvignon grape extracts [48,49], and black rice bran [50].

**Figure 4:**
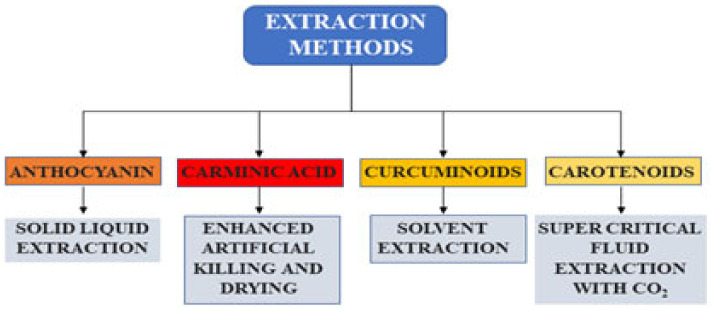
Extraction methods for the recovery of natural colorants.

The process parameters for the extraction of anthocyanins were optimized from plant tissues, food products, and waste sources. Several modern technologies, such as pressurized liquid extraction, supercritical fluid extraction, ultrasonication, and pulsed electric field extraction, have emerged to recover anthocyanins. These techniques observed high yield with increased mass transfer, less processing time, and low energy requirement. The application of eco-friendly solvents is another alternative to conventional solvent extraction. Precipitation, membrane separation techniques, solid phase extraction, and chromatographic techniques are also employed for the concentration and purification of anthocyanins. A solvent system composed of methyl-t-butyl ether, n-butanol, acetonitrile, and water, was proposed to separate anthocyanins from a wide range of food materials. The anthocyanins were extracted with 0.15% HCl in methanol from oxalis leaves. The separated anthocyanins are unstable and undergo degradation. The structure, pH, temperature, oxygen, and enzyme are the important factors that influence the strength of anthocyanins. To increase the stability of anthocyanin, the structure of the anthocyanin can be changed into aromatic rather than aliphatic. To extend the applications of food colorant, the acyl bunches with higher hydrophobicity, increasing the anthocyanins’ strength. Spray drying, freeze drying, encapsulation, and coatings enhance the color of anthocyanins. Encapsulation of polysaccharides even increases the delivery in the gastrointestinal region. Extraction methods available for the recovery of anthocyanins from natural sources are presented in Table 1. In addition, a few of the extraction methods of natural colorants are illustrated in Figure 4.

### 5.2. Carotenoids

The carotenoids are the derivatives of the C40 tetraterpenoid pigment phytoene. The basic structure can be modified by hydrogenation, dehydrogenation, cyclization, and oxidation. Carotenoids can be classified as provitamin A and non–provitamin A carotenoids [59]. In colored fruits and leafy vegetables, microalgae are the main natural sources of carotenoids. The fresh leaves of sweet potato, spinach, and turnip greens are major sources of lutein and zeaxanthin. Tomato is an abundant source of lycopene. Dark red fruits are established as a rich source of lycopene. Astaxanthin is high in the red lobster (*Panulirus argus*) (7.73 mg/100 g). Shrimp showed a carotenoid content of 9.93 mg astaxanthin/100 g oil. The root crab (*Goniopsis cruentata*) showed 17.7 mg/100 g astaxanthin content, and the Guaiacum crab (*Cardisoma guanhumi*) 2.11 mg/100 g. Carotenoids are recovered by solvent extraction [60]. The solvents used in the processes should be eco-friendly, commonly from renewable resources of biomass feed. The extraction process’s efficiency depends on the solvent’s carotenoid composition, temperature, and nature. Acetone, ethanol, and hexane are frequently used as extraction solvents. The Soxhlet extraction technique yields the highest recovery of carotenoids [61]. The feed material is finely ground and stirred with solvent in a nitrogen atmosphere. The homogenate is filtered, and the residue is extracted [62]. The extracts are combined and concentrated by rotary evaporation. This method is appropriate while using green tissue of plants, flowers, and fruits for extraction. However, supercritical fluid extraction (SFE) using CO_2_ as a solvent and ethanol as a co-solvent also has significant and selective extraction of carotenoids [63]. Ultrasound-assisted extraction (UAE), pressurized liquid extraction [64], pulsed electric field (PEF) extraction, and enzyme-assisted extraction (EAE) are the other non-conventional techniques for the extraction of carotenoids [65]. The saponification is carried out by adding 15% KOH to the extract. This saponification can be performed by heating the alkaline mixture in a water bath for 10–15 min in a nitrogen atmosphere for 10–15 h [66]. A mechanical disruption is required for efficient separation to extract carotenoids from algae. Carotenoids are extracted from algae with more polar solvents [67]. Carotenoids are transformed into vitamin A, with variable degrees of conversion efficiency. Carotenoids, along with vitamins, are chemopreventive agents that act as antioxidants. β-carotene is an orange pigment associated with protection against heart disease and cancer. Studies show that the consumption of β-carotene-rich foods significantly reduces lung cancer. Lycopene is the best biological suppressor [37,68]. Lutein and zeaxanthin are stored in our body in the retina. This xanthophyll has antioxidant power more than ten times greater than β-carotene and 500 times greater than vitamin E. Astaxanthin is a pigment found in aquatic animals [69]. Some of the commonly employed methods of extraction of carotenoids are presented in Table 2.

### 5.3. Carminic Acid

Carmine is the water-soluble food colorant used in yogurt, juices, and ice creams. The colorant is produced from carminic acid, abundant in female cochineal insects (*Dactylopius coccus*). The extraction of carmine would be a complex aluminum/calcium chelate of carminic acid from dried cochineal [78]. The extraction yields 65% of the carminic acid-producing carmine. The color of the product depends on the proportion of aluminum and calcium. Around 10^5^ insects are required to yield 1 kg of dried cochineal. The separation process involves slaughtering and drying the insect bodies. Solar drying of the insects provides silver cochineal [79]. Carminic acid is 7-α-D–Glucopyranosyl-9,10–dihydro -3, 5, 6, 8 tetrahydroxy-1-methyl-9,10-dioxo2-anthracene-carboxylic acids. It competes with betanin and anthocyanins in food coloring [80]. Wild-type insects contain at least 16% (by weight) carminic acid. Carminic acid found extensive applications in beverages, cake dry mixtures, and conserves. It also finds its application in the pharmaceutical, cosmetic, and textile industries. Carminic acid reacts with ammonia to obtain acid-stable carmine [81]. High-grade cochineal is retained by 10 to 14 sieves. The raw material is cleaned with hexane to remove waxes and grease. The dried feedstock is milled to 100 mesh to break down the tissues containing the colorant. The dried ground cochineal is fed into a reactor containing boiling sodium carbonate. The boiled carminic acid solution is then filtered. Precipitation of carmine is then achieved by reducing the pH from 9 to 5 by adding citric acid or tartaric acids. Sedimentation of the carmine occurs by gravity. The batch cycle times are 4–6 h. The washed product is sterilized at 120 °C, then dried under partial vacuum. According to the FDA, carmine contains 50% (*w*/*v*) carminic acid, pH 5.0–5.5, total solids: 5.7–6.3%, total protein: 2.2% max., lead: 10 PPM max., and arsenic max. 1 ppm.

### 5.4. Curcuminoid

The recovery of curcuminoids from *Curcuma longa* rhizomes was executed by solvent extraction. Curcuminoids are hydrophobic yellow pigments [82]. They have certain antioxidant and anti-inflammatory effects. Curcuminoids are used in foods and are obtained industrially from turmeric with solvents such as ethanol. Curcuminoids have three basic constituents, namely, curcumin, demethoxycurcumin (curcumin II), and bisdemethoxycurcumin (curcumin III), which belongs to the diferuloylmethane group of phenolic compounds [83]. Curcuminoids found extensive application in the pharmaceutical industry to treat cancer, oxidative stress, and diabetes [84]. Conventional techniques for the extraction of curcuminoids involve solvent extraction and steam distillation. Various advanced techniques, such as supercritical fluid extraction [85], microwave-assisted extraction [86], ultrasound-assisted extraction, and enzyme-assisted extraction, have recently emerging for the extraction of curcuminoids from natural resources. Three-phase partitioning techniques involve ammonium sulfate and tert-butanol added to the raw material [87]. This technique is a simple theory with recent application in separating proteins. Enzyme purification, such as peroxidase, β-galactosidase, and invertase, extracts natural products from the oleo gum resin of *Boswellia serrata* [88].

## 6. Major Applications of Natural Colorants Established So Far

Anthocyanin, a natural-food colorant with a plethora of health and nutritional benefits, will be supported through viable technological points to food systems. Consumer demand for food products with natural food ingredients and colorants has resulted in the global demand for anthocyanin. Overall, health and nutrient supplements require versatile qualities, and anthocyanin is one of them. China has recently suggested a daily intake of 50 mg, 12.5 mg/day in the US, Europe (19 to 65 mg/day—men; 18 to 44 mg/day—women), Australia (24 mg/day), and Finland (150 mg/day). Daily intake may protect from neurodegenerative and cardiovascular diseases, and attempts with chemical modifications such as acylation result in more stable anthocyanins [89]. Another prominent feature of anthocyanin is its antioxidant property. Anthocyanins (blue, red, or purple pigments) are found in flowers, fruits, and tubers. Cyanidin-3-glucoside is the major form of anthocyanin found in most plants, and its color and stability are affected by pH, light, temperature, and structure. Anthocyanin has a wide range of color shades that depend on the food products’ molecular structure and pH value. Researchers utilized the color change behavior under different pH (acidic-red pigment) and blue color in alkaline pH conditions. These properties are explored in smart packaging industries, where film color change indicates the quality/freshness of the packaged food in real time. A pH-responsive color-changing attribute of anthocyanins is used in smart packaging that helps to monitor foods such as milk, meat, and fish [90]. The colorant has good antioxidative and antimicrobial activities that prevent the occurrence of non-communicable diseases. Blackcurrant extracts are intensely colored due to the presence of anthocyanins and have the potential to act as natural colorants in various consumer products. Different natural colorants that have been commercially exploited in USA and EU are anthocyanins, carotenoids, and curcuminoids from turmeric (*Curcuma longa* L.), and carminic acid from cochineal (*Dactylopius coccus*) extract. Anthocyanins from flowers and bracts of *Thymus moroder* are used for coloring and fortifying antioxidant foods and yogurts’ applicability for natural colorants to commercialize novel foods [91]. ACN also proves to be a safe and potent poultry feed with growth, productive performance, organ function, and reduction in pathogenic bacteria in the gut and cloaca. Further, in-depth research is required to unearth the mechanisms of action that take place at the molecular level to confirm pharmacological and growth promotion in different poultry [92]. Since ancient times, dyes from natural resources have been proven to be eco-friendly. This colorant is known to protect plants in extreme weather conditions, which can be used to develop super cloths. Since the world is moving towards ‘Eco-fashion’ as visual markers, solar dyes will increase fabric output, reduce total cost, and have good washing fastness in anthocyanin-dyed fabrics. In addition, it can convert light energy into electrical energy, which is a source of organic solar cells [93]. Beneficial properties of anthocyanins include diabetes prevention, protection from skin aging, cancer, and damage induced by ultraviolet rays, anti-fatness, and oxidative stress effects of anthocyanins. In addition, researchers from the UK have claimed anthocyanin’s key benefits in improving arterial stiffness (cardiovascular disorders). They found that the intake of ACN-rich purple potato consumption systematically reduced pulse wave velocity (PWV). Some key applications of natural colorants in the food industry are listed below (Table 3). A few of the applications of colorants as food markers and nutraceuticals, which have greater scope in the future, are discussed in the section below.

### 6.1. Nutraceuticals

Carotenes are exploited as a nutritional supplement because of their bioactive compounds [28]. Colorants with strong pharmacological significance include flavonoids and tannins [26]. Because of their potential health benefits as natural antioxidants, interest in anthocyanin food colorants is increasing [101,102]. Anthocyanin isolates, for example, may operate as a product testing marker for food; increase the nutritional content of foods and beverages; and play a key role in lowering the risk of cardiovascular diseases, cancer, and cerebrovascular diseases [103,104]. In addition, many natural colorants, namely, carotenoids, and chlorophylls, are suggested as nutritional supplements from an early age among humans in light of their functional ingredients. 

### 6.2. Food Safety Markers

Natural food colorants find applications in intelligent packaging where they detect the change in chemical and physical properties of packed foods. There is a surge of such applications in the current decade. Under the prevailing monitoring system, a substantial volume of food is wasted based on established quality control protocols based on expiry dates [105]. This intelligent technology was worth USD 17.5 billion in 2019 and is estimated to increase with an annual growth rate of 6.78 percent to USD 251.6 billion by 2025. Anthocyanins exhibit a red hue at below 4, a purple hue at pH 7, a deep blue hue at pH 8, and a light yellow hue beyond pH 8 [106]. They are susceptible to variations in physical and chemical changes in packed foods, making them a smart sensor of the shelf life of packaged foods. Similar to anthocyanins, alizarin, curcumin, and betalains have similar properties and are prospective candidates as food safety markers in intelligent packaging [107]. 

Natural colorants from hibiscus supported on chitosan starch films were used for packing chicken. As the pH rose, the films changed color from purplish-grey to a dark and green hue due to the build-up of ammonia and amines produced by mesophilic bacteria spoiling the meat [108]. Another research reported on color change from pink-colored anthocyanins derived from *Lyciumruthenicum* on cassava starch to gray and greenish-yellow, indicating the spoilage of pork when pH changed from 2 to 13 [109]. Similarly, natural colorants have been successfully used for the intelligent packaging of meat products such as beef, pork, and poultry [110,111,112,113,114,115,116,117,118], seafood and fish products such as shrimp and fish fillets [119,120,121,122], and milk and dairy products [123,124,125,126].

## 7. Impact of Synthetic and Natural Colorants

Awareness of synthetic food colorants has increased because of the escalating health issues, even in toddlers, with unhealthy food. Regulatory authorities have been established to streamline the safe food colorants added to food and periodically review the food colorants. The discussion on the Southampton study and the conclusion by the FDA prompted certain consumer groups and enterprises to demand that artificial food colors be replaced with natural colorants, which had a significant impact on the growth of the natural food colorant industry [127,128]. Some textile colorants, such as triphenylmethane and azo dyes, are synthetic food colorants [129], which should serve as an eye-opener to consumers. The WHO and FAO have created a database for worldwide reference, a compilation of existing evidence concerning the biological activity of food additives, along with The Codex Alimentarius collection [130]. Food colorants impact health and the environment, which is discussed in this section, and there is a requirement to find alternatives to harmful synthetic colorants. 

### 7.1. Health Impacts

Toxicity effects are assessed in six distinct species, of which three were mammals due to their similar physiology to humans and NOAEL (no observed adverse effect level). ADI is evaluated based on experimental studies [130,131]. Some synthetic colorants that are harmless become harmful after digestion of the food product due to metabolism and bio-accumulation due to their low degradable nature. Due to customers’ health complications, the U.S. has restricted water-soluble synthetic colorants to seven, India to six, and the EU to seventeen [127,132]. Several health problems, such as allergies and hyperactivity, have been linked to food colorants, especially azo varieties and nitrous derivatives such as E102, E110, E122, E123, E124, and E129 [133,134,135]. In 2007, the usage of E128 was banned due to factual data on its carcinogenic potential (Commission Regulation (EC) No. 884/2007). The Southampton report on the usage of synthetic food colorants on children aged less than ten years found the susceptibility of children to hyperactivity, which led to the EU requiring a warning message on food packets containing the synthetic colorants [136] from July 2010. Six synthetic colorants confirmed to cause hyperactivity in children are carmoisine, Allura Red, ponceau 4R, sunset yellow, tartrazine, and quinoline yellow [136,137]. Another study on the effect of synthetic colorants in beverages on children aged five found a link between colorants and attention deficits [138]. The effect of synthetic colorants on female rats in the prenatal and perinatal periods showed impairment of cognitive characteristics due to mutation in their babies [139]. Blue hues E131 and E133, used in lollipops, can infiltrate epithelial cells and reach the circulatory system without going through the digestive system, making them more harmful since their chances of degradation becomes negligible [140]. Regular consumption of tartrazine, which is commonly used in beverages, candies, and ice creams, has been linked to hyperactivity and obsessive-compulsive disorders in toddlers by causing DNA damage by interaction with human serum proteins [133,134,141]. Various investigations of tartrazine have demonstrated possible cytotoxicity [142], genotoxicity [143], and neurotoxicity characteristics [144]. Prolonged tartrazine consumption is believed to reduce liver functionality in postmenopausal women due to its impact on the female hormone estrogen [145]. Allura Red AC has been determined to be dangerous to human health at high dosages [130]. Red 2G was banned in 2007 since it was found to produce aniline while cooking [96]. Triphenylmethane dyes, which are illegally added as a food colorant in fish, were found to cause carcinogenic and genotoxic health issues [146]. Yellow colorants such as sunset and quinoline yellow have shown genotoxic characteristics [147,148]. Both are inclined to bind to protein molecules, especially cow and human cells [149,150]. Assimilation of a high dose of patent blue led to a decrease in hemoglobin, red blood cell counts, and hematocrit [151]. The synthetic pigment erythrosine was found to induce thyroid tumors in rats [152] and cytotoxicity in lymphocytes in humans [142]. According to a recent investigation, indigo carmine might lead to hypotension [153]. In vitro experiments on the influence of brilliant blue on serum and lymphocyte cell lines have revealed that this dye has mutagenic and carcinogenic properties [148] along with thinning of the retina [154]. Brilliant black had minimal genotoxic effects on human lymphocytes and *Vicia faba* [155]. Sudan I dye is a banned class 3 carcinogen (IARC, 1975), which was usually used for coloring curries, chili powder, and paprika. Various research reports confirm the detrimental effects of synthetic colorants from acute to chronic levels.

On the other hand, natural food colorants have very few toxicological studies reported, and only when they are adulterated with other synthetic food constituents. Adulteration is rampant with synthetic colorants as well. Since natural food colorants are mostly derived from food materials that are safe to consume, it is usually generalized to be safe and is exempt from USDA certification. Future studies on individual natural food colorant or their mixtures can probably throw light on their toxicity, and currently, significant restrictions are prevalent due to ethical, legal, and social issues.

### 7.2. Environmental Impacts

The toxicity assessment focuses solely on laboratory-based toxicity assessments of new dyes, ignoring the repercussions of prevalent degradation compounds. The toxicological character of dyes in the environment is determined by extrinsic variables such as physicochemical/environment and intrinsic variables such as interaction effects and metabolism of dyes and microorganisms [156]. Mostly, all azo dyes, brilliant blue, erythrosine, and green, are negligibly absorbed by the human tract and are found unchanged in human excreta [130]. This leads to new emerging pollution since erythrosine is a common colorant in pharmaceuticals. Mostly, these colorants exhibit carcinogenic and genotoxic characteristics.

Moreover, specific environmental mechanisms can cause non-toxic colorants to become hazardous metabolites under certain circumstances, such as hydrolysis of some azo dyes, such as reactive black 5, which yielded vinyl sulphone and aromatic amines [157,158]. Most of the colorants are water soluble making isolation and treatment impossible and have been prevalent in the environment for many years [159]. These residual colorants impact the aquatic environment greatly, which is evident in the frequent death of fish and aquatic plants. The degradation of dyes also varies from one ecosystem to another, developing habitat microheterogeneity [160,161]. Wetlands with a predominantly anaerobic environment, such as marshes and paddy fields, may enhance the azo bond breakdown and serve as reservoirs for highly poisonous aromatic amines, which will affect living organisms in such ecosystems [156]. Aquatic ecosystems have been affected by the degradation of azo dyes into poisonous amines and hydrazines [162]. At subsurface soil levels, humic layers cause the photodegradation of dyes [163]. A few examples of predominant effects on aquatic organisms are a toxicity increase in *Vibrio fischeri* due to hydrolysis of reactive black 5 [152] and Procion red MX-5B, which, in turn, affected *A. salina* larvae [164]. Bismarck brown Y and acid red 97 affected embryonic development in frogs [165]. Plant growth was affected by reducing chlorophyll, carotenoids, carbohydrates, and protein contents [166]. Biomagnification of dyes, especially azo dyes, on prolonged exposure causes mutagenic and genotoxic effects in humans [159,167]. It can be inferred that these degraded products of dyes ultimately reach humans through the food chain from both biotic and abiotic components of the environment and are comparatively more harmful to humans than the environment. 

On the other hand, natural colorants are biodegradable and non-toxic and impart anti-carcinogenic and anti-microbial characteristics to their constituent food matrix [167,168]. The impact of some natural food colorants is represented in Table 4.

## 8. Challenges in the Sustainable Utilization of Natural Colorants

Natural food colorants have restrictions due to their poor thermal stability, possible interactions with other food components, and toxins because of coproduction and high-cost index. Moreover, their yield is comparatively less, and possible remedial measures are suggested to overcome the challenges of commercialization and sustainability.

### 8.1. Colorant Stability 

Most natural food colorants currently in use are highly susceptible to temperature, oxygen degradation, light, and pH changes [189]. During the storage and transport of food colorants, they may be exposed to air, heat, and light, resulting in pigment loss over time [190]. Carotenoids are more thermally stable than anthocyanins and betacyanins, which have low thermal stability. Natural colorants tend to lose their original color and are susceptible to color change when heated. For example, betacyanin extracted from *Opuntia* fruits and red beet could only retain 12.5 and 1.7 percent of their initial absorbance at 535 nm, respectively, when heated to 90 °C for 6 h. This led to a loss of red color. The most thermosensitive was the *Opuntia* fruit active constituent, which degenerated by 58 percent at 50 °C, 91 percent at 70 °C, and more than 98 percent at 90 °C after 6 h, whereas red beet had a half-life of 1.84 at 90 °C [191]. The discrepancy in degradation rate between red beet and *Opuntia* extracts could be due to the chemical configurations of the constituent betacyanin food colorants prevalent across both extracts. Furthermore, the existence of other chemicals that could alter consistency during heating was in agreement with earlier research [192]. Anthocyanin food colorants vary from purple to blue at high pH and from red to pink at low pH values when the pH of the food matrix changes [193,194]. Stabilizers may be added to the final food colorant composition to improve its stability [174,195].

Advanced processing techniques can eliminate the need for stabilizers making them wholly natural. For example, in comparison to a spray-dried colorant, an advanced technique such as freeze drying could retain the color better [196]. Moreover, freeze-drying eliminates the need for additional stabilizers [197,198]. Additionally, to extend the shelf life of natural colorants, contemporary processing like microencapsulation, emulsification and irradiation, and packaging strategies, can be implemented [176,181,199]. Encapsulation can greatly prevent the degradation of color [197,199,200,201,202,203]. Great care must be emphasized in product testing and evaluation.

### 8.2. Legal Regulations 

Regulations on the usage of food colorants are usually country specific. Three synthetic colorants that are legal in the US are not allowed in the EU. In contrast, nine of the fifteen synthetic food colors that are legal in Europe are not approved in the US. Likewise, eight synthetic colorants are permitted in India, of which one is not approved in Europe. Food exporters and importers are perplexed since the same food color may be allowed in one country but banned in another [127]. Nevertheless, the colorants proposed by the FDA are the standard for drafting individual countries’ safety norms on food colorants. The United Nations confirmed the compliance of anthocyanin and lycopene production to human rights (UN, 2015). Phycocyanin, a pigment found in spirulina species discussed earlier in this review, is the most stable natural colorant for the blue hue. Yet, it is not permitted as a raw material for colorant in the US and EU. Spirulina sp. is categorized as cyanobacteria by the FDA and is considered a food rather than a source of colorant; hence it does not comply with regulation 21CFR 73.260 [132]. Due to the contradictory restrictions placed on certain natural colorants in various countries, the approval and commercialization of safe and new colorants are usually delayed. Hence, a common regulatory authority can issue international standard codes for natural colorants to ensure consumers’ safety and health.

### 8.3. Cost Component

The commercialization of many natural colorants is offset by their cost due to extraction, purification, and isolation procedures compared to their industry counterparts; compared to their synthetic counterparts, energy, and water consumption are relatively higher for natural colorants [35]. Besides, they must be added more than synthetic colorants to obtain a satisfactory hue [190,204,205]. Nevertheless, they can be valorized to yield animal feed or processed further to obtain colorless phenolics and pectic oligosaccharides [177,206,207], which can reduce the overall cost of production of natural colorants. Energy-intensive technologies, recycling, and reusing solvents can be encouraged as their sale can reduce the cost of food colorants [208,209]. More intensive research is required to make natural colorants cost-effective and to realize a circular economy shortly.

### 8.4. Yield

Natural food colorants are mostly derived from biotic components, while synthetic colorants are derived primarily from abiotic components. Yield is calculated by pure food colorants’ yield from the original raw materials. The yield of anthocyanin colorant extracted from grapes ranged between 30 and 750 mg/100 g. The amount of lycopene extracted from fresh tomatoes varied from 1 to 20 mg/100 g. Furthermore, 24 and 26% carminic acid is extracted from *Dactylopius coccus* Costa [210], whereas 90% yield could be achieved in the case of azo dyes [210]. Hence, to increase the yield, sources that are richer in pigments and new breeds of commercially used plants should be produced [211,212]. Technologies such as supercritical extraction, encapsulation, microextraction, ultrasound, and microwave-assisted extraction are environmentally friendly and help to attain higher yields [128,213].

### 8.5. Co-Production of Toxins

The production of food colorants from fungi has shown toxicity by the coproduction of a metabolite harmful to the human liver. This can be overcome by following, generally recognized as safe (GRAS) protocols for producing food colorants from *Aspergillus niger* [214]. Metabolic engineering can also be used to avoid the coproduction of mycotoxins along with pigment production. Inactivation or enhancement of selected steps of a biosynthetic pathway by a chemical approach can be an alternative tool to metabolic engineering, using mutations or genetic transformation techniques to suppress toxin coproduction [214].

### 8.6. Future Prospects

Synthetic colorants are becoming less popular all over the world, with Europe, Asia, Mid East, the US, and Africa reporting higher levels of avoidance, paving the way for natural food colorants to have a significant market [215,216,217,218]. Currently, natural colorants contribute about 36% of the global market for food colorants [28,181,219]. The U.S. Food and Drug Administration’s restriction on FD&C Red No. 2 (amaranth), Scarlet GN, and Ponceau GR in France and Orange RN in the United Kingdom has resulted in an upsurge in demand for natural sources of red food colorants [220]. In the EU, 13 naturally produced colors are permitted, while in the US, 26 colors are exempted from certification [132]. With biotechnological advances, contemporary researchers can simplify the techniques for producing the existing colorants and find new colorant sources for their commercialization [221]. The principal markets of food-grade bio-colorants are in the US, EU, Japan, and emerging markets in China, India, and South Korea. Developing countries such as India and China may play a major role in supplying natural colors either in processed forms or as raw materials to the EU markets due to their favorable climatic and production conditions coupled with the rise in their middle-income families. More research in support of safety in food products could influence US, Asian, and EU regulations. Consumer awareness of the multifaceted benefits of food colorants will encourage the commercialization of safe and novel food colorants. Moreover, consumer support in terms of compromising on cost and stability to industries that resort to natural food colorants will enable rapid acceptance. Many consumers are unaware that Nestle replaced their blue colorant in candies with a color extracted from *Spirulina* in 2008 [222].

In the future, wastes derived from the processing of food products will attract more scientists as they act as a desirable source of natural food colorants [223]. For instance, novel extraction methods have been used to remove beta-carotene from tomato waste, lycopene from tomato waste [224], and lycopene from papaya waste. In addition, gac fruit has been studied as a great source of lycopene and beta-carotene. Further, a new bright yellow dye, which is highly water soluble, has been extracted during the production of apple juice and apple cider. This acts as a prospective natural alternative to tartrazine. Besides, microalgae are developed as a novel source of food colorants as various chlorophylls are available in most microalgae. Furthermore, microalgae are a rich source of beta-carotene that is found in green microalgae (Chlorophyta), brown microalgae (Diatomophyceae), cryptomonads (Cryptophyta), euglenoids (Euglenophyta), and dinoflagellates (Dinophyta). Light and temperature are the prime factors for consideration, while we expect a high production rate of colorants from microbes as they are generally required for biomass growth. The suitable temperature for the production of pigments from most of the microalgae species lies in the range between 25 and 36 °C. Yet, high temperature (55–60 °C) is preferred for producing total carotenoids from green microalga. It is also noteworthy to consider that the concentration of pigment in microbes is greater than that present in vegetable or fruit sources. For example, the concentration of chlorophyll is found to be 20% more in *A. platensis* biomass than in spinach [99,225]. There is an enormous source of dyes forming plant varieties, mineral constituents, and animal species. Hence, by advanced methodology, either in cultivation or processing, maximum production rates can be delivered. The usage of insects and microbes for colorant formation can be solely explored. The application of naturally made colorants can be accounted for in the performance improvisation among various colorants. Several applications can be made on par with current trends, such as dye-sensitized cells, anti-cancer activity, biosensors, cell-imaging techniques, and corrosion inhibition mechanisms, can also be highlighted. It is mandated to conduct enhanced research activities for optimizing colorant production from various natural origins.

## 9. Conclusions

Technological advancements in extracting natural colorants hinder their use in everyday food products. This opens the door for alternative sources of colorants, which would be more stable and physiochemically feasible to extract. Some fruits, such as Andean, Amazonian, and other South Asian fruits, are claimed to be underutilized raw materials for extracting natural food colorants. Besides, upcoming technologies, namely, ohmic heating and enrichment factor-based technologies, employ less energy and water to remove colorants, resulting in a high recovery rate. For compounds that are heat sensitive, the high-pressure extraction factor favors the extraction without induced temperature, thus preserving the functional characteristics of colorants. Though alternative sources for natural colorants have gained interest, optimization is required to produce eco-friendly colorants with cost-effective features. Besides, optimization is also necessary when nutrient-rich additives are added to the colorants, which might affect the health benefits of food products. Thus, with optimization, nutrient-rich additives can be safely added to the colorants that enhance the food products’ overall properties without affecting their texture and sensory outcomes. More research is required to assess the chemical composition of food colorants derived from natural sources. Furthermore, the stability of the food colorants has to be considered when they are used in food products with varied pH and temperature ranges.

## Figures and Tables

**Table 1:** Most common extraction methods for the recovery of anthocyanins from natural sources.

Source	Method	Reference
Blueberry	Solid-liquid extraction	[51]
Blackberry	Solid-liquid extraction	[52]
Jamun	Solid-liquid extraction	[53]
Red radish	Solid-liquid extraction	[54]
Jambolan	Supercritical fluid extraction	[55]
Blueberry	Supercritical fluid extraction	[56]
Cherry	Ultrasound-assisted extraction	[57]
Strawberry	Ohmic heating-assisted extraction	[58]

**Table 2:** Most common extraction methods for the recovery of carotenoids from natural sources.

Source	Method	Reference
Buriti (*M. flexuosa*) pulp	supercritical fluid extraction with CO_2_	[70]
Microalgae	supercritical fluid extraction with CO_2_	[71]
Tomato	supercritical fluid extraction with CO_2_ + ethanol	[72]
Carrot	supercritical fluid extraction with CO_2_ + vegetable oils as co-solvent	[73]
*Pandalus borealis*	Solvent extraction	[74]
Tomato	High-pressure extraction	[75]
Gac fruit	Enzymatic extraction	[76]
Biowaste (peels, seeds)	Ultrasonication	[77]

**Table 3:** Applications of natural food colorants, their sources, and extraction procedure.

Product	Pigment Origin	Extraction Procedure	Applications	Reference
Bakery products				
Cupcakes	Roselle (*Hibiscus sabdariffa* L.)	Roselle calyces (28 °C, 3 h) were dried, after which it was ground (0.55 mm) and soaked in water (200 mL). The resultant solution was heated at 80 °C for an hour.	Enriches the composition of cupcakes and improvises preservation of cakes.	[94]
Cakes and cookies	Tomato wastes	Lycopene was extracted from tomato wastes when heated at 20, 30, and 40 °C with a time frame of 15, 30, 45, and 60 min. The extraction solvent has to be removed to use the lycopene.	Increases antioxidant properties and cake volume.	[95]
Wafers	*Arbutus unedo* fruit	Extraction is carried out using the application of heat. The recovery rate was 60% of the total dry fruit weight.	The properties of the wafers change after a storage period of 3–6 days.	[96]
Alcoholic beverages	*Porphyridium sp.* microalga	Centrifugation, microfiltration and freeze-drying techniques are carried out for the extraction process.	Imparts yellow color to the food products.	[97]
Condensed milk-based confections and doughnut icing	*Ficus carica* and *Prunus spinosa* L.	Peels and epicarps were treated by freeze drying and milling, followed by an ultrasound to extract the colorant.	They are applied in milk-based products.	[98]
Ice cream	Microalga (*Nannochloropsis oculata, Porphyridium cruentum,* and *Diacronema vilkianum*)	Spray drying and centrifugation techniques were used to extract the colorant.	The colorant imparts pink and green colors to the ice cream depending on the nature of the microalgae.	[99]
Sausages	Brown seaweed (*Cystoseira barbata*)	Drying, milling and aqueous two-phase extraction techniques were used to extract the colorant.	The pigment imparts red color to the food products.	[100]

**Table 4:** Some of the natural food colorants, their sources, along with their beneficial properties.

Food Colorant	Source	Benefits	References
Carminic acid -hydrophilic	Animal-based	Prevention of diabetes and cancer. Exhibits antifungal and antioxidant properties.	[169,170,171]
Anthocyanin -hydrophilic	Plant-based	Prevention of cardiovascular diseases, cataracts, Alzheimer’s disease, and cancer. Management of obesity and diabetes mellitus	[16,136,172,173,174,175,176,177]
Curcuminoid -lipophilic	Plant-based	Prevention of cancer. Exhibits anti-bacterial, anti-inflammatory, antifungal, and antiseptic properties. Possess excellent wound-healing characteristics	[178,179]
Carotenoids -lipophilic	Plant-based	Antioxidant and dietary source of vitamin A prevents cardiovascular diseases and cancer	[173,177,180,181]
Chlorophyll -lipophilic	Plant-based	Prevention of cataracts, colon cancer, coronary heart disease, and diabetes	[182,183,184]
Betacyanins -hydrophilic	Plant-based	Prevention of cancer, high-density lipoproteins, and cardiovascular diseases	[185,186,187,188]

## Data Availability

Data is contained within the article.
